# Through the mists of time: *Sushrutha*, an enigma revisited

**DOI:** 10.4103/0970-0358.59285

**Published:** 2009

**Authors:** Philip Philip Puthumana

**Affiliations:** Department of Plastic and Microvascular Surgery, Little Flower Hospital and Research Centre, Angamaly, Kerala, India

**Keywords:** Bower manuscript, history of plastic surgery, *Sushrutha*

## Abstract

*Sushrutha* had been viewed in textbooks of plastic surgery as belonging to the caste of potters who performed surgery in India. We have examined the available source documents and other references to the technology of the period to examine this assertion and are convinced that there is no evidence to support this. The period, technology and geographic references in *Sushrutha Samhitha* are correlated with settled positions on these to arrive at an understanding of the time and knowledge which is described. Source of erroneous interpretation of *Sushrutha* as a potter is also examined and clarified.

## INTRODUCTION

The introductory paragraph on ‘History of Plastic Surgery’ in the text book - Plastic Surgery 2^nd^ edition, profiles *Sushrutha* as belonging to the caste of potters and further asserts that the caste of potters traditionally performed surgery in India. (*Mathes, chapter 2).*[1]

This article is a journey of discovery triggered by this off hand comment in the premier reference book on plastic surgery in modern medical literature and an attempt to set the records straight.

Looking at *Sushrutha,* we are faced with vast distances over time. The main questions that arises are: who, where; when and what remains to tell us today the true story?

History is often said to be the stories told by the victors. And rulers are notorious through history to re-enumerate history to justify their current positions and actions. Multiply it with thousands of years and the issues get muddled up further and make the reality still unclear.

Before examining *Sushrutha*, we need to understand briefly how human civilization evolved. Observation of patterns and planned and structured methods to transfer the observations with inferences to next generation lies at the root of our progress.

While the documentation of treatments exist in the Vedas, the two epics of Indian culture Ramayana and Mahabharata, refer to organisation of treatment in military campaigns and is often painted in flowery language and exaggeration abound. In Ramayana there are references to an organised system of treatment, with the description of an all rejuvenating herbal medication ‘sanjeevani’ and the search for this herb. Later, in Mahabharata are what can be viewed as earliest references to external fixation of fractures as narrated by a lay person on ‘the bed made of arrows so that severely injured Bhishma can rest till death takes him’.[2–4]

In the well planned settlements of Harappa itself there were special areas deduced to be hospital or treatment buildings. These make us think that there was an organized system of medical practice carried on from the earliest times, and Sushrutha Samhitha was but one text book used for training of the surgeon.[5]

## MATERIAL AND METHODS

*Sushrutha Samhitha* as a key text book for the development of surgery in modern times, carried through the translations into Arabic as Kitab-i-Susrud in eighth century A.D. on orders of the Caliph Mansur and from there to Latin, is documented by none other than Sir William Harvey.[6–8]

This elucidates the fact that *Sushrutha Samhitha*, as a Bible of surgery in those times, was studied by students of medicine for nearly two thousand years, much like medical students of today study ‘Bailey and Love's Textbook of Surgery’.

But the only conclusive text available in a written form of *Sushrutha Samhitha* is the Bower manuscript. The translation of *Sushrutha Samhitha*, The *Sushruta Samhita*: An English Translation Based on Original Texts, by Kaviraj Kunjalal Bhishagratna is the root text examined. Available literature on the subject and allied areas were also perused. Correlations with other areas referred in *Sushrutha Samhitha,* like metal work, geography and other period references, are used to establish the location and period where the source manuscript was written. Reasons for wrong interpretation of the history of surgery in India are discussed based on the established commentaries.

## DISCUSSION

The *Bower Manuscript* is a Sanskrit-language manuscript written in the Brahmi Script. Lieutenant H. Bower got the manuscript in 1890, in Kuchar, in Eastern Turkestan. It was then sent to Colonel J. Waterhouse, who was the then President of Asiatic Society of Bengal. Dr. Augustus Hoernle deciphered the manuscript.[9,10]

This manuscript written in the Brahmi script was the corner stone in dating the times of Sushrutha. There are other records, especially of translations into Arabic by Ibn Abi Usaybia (1203-1269 AD).[11]

Ayurvedic literature is preserved almost exclusively in the Sanskrit language, and originally in the form of manuscripts written on birch bark, palm leaves or paper. India has, over the millennia, developed more than a dozen different alphabets. The scribe who copied out the manuscripts would use the script that was local to the place of work. So it is quite normal to find Sanskrit medical manuscripts from Kerala in the Malayalam script, while a manuscript of the very same text copied in Bengal would be in the Bengali script. Both manuscripts would still be in the Sanskrit language and would be virtually indistinguishable if read aloud.[7] This system of transmission resulted in the creeping up of errors. More over the information needed an interpreter to transmit the meaning to the student. The teacher, who does this function, imparts his own inferences, mostly influenced by the changes and advances around him and his individual perceptions.

This influence is well evident in the English translation of *Sushrutha Samhitha* by Kaviraj Kunjalal Bhishagratna, who wrote the first translation in 1907. In the introduction, he not only tries to explain the level of understanding that this document had, as equal to, if not superior to the expanding scientific understanding at the turn of the twentieth century, but also ends the introduction with a prayer to the government to support its regeneration.[12] Various future narrations on Ayurveda and other alternate systems in popular media try to build on this concept. Thus is created the current schisms in treating the diseased human being and in development of health policies.

The information contained in the text that helps to fix the approximate date is regarding the materials used to fashion surgical instruments and the techniques of fabrication. There are very detailed descriptions of various instruments in *Chapter VII. Yantra-Vidhimadhyayam (Surgical Appliances, their Uses and Construction.).* Detailed instructions are given on the fabrication of more than 100 instruments in iron. But he also suggests that other materials like ivory can also be used for fabrication of some of these instruments. (But the diagrams of these instruments that were available in some publications cannot be conclusively proven to be of this period and as documented in the original text based on available references, in the absence of ability to observe the source document. This is stated here to give a proper perspective, as in the case of other images, which were works of a much later period, the diagrams of these instruments could also be from a later period.). The description of two blades of a ‘*Swastika*’ welded together by means of a bolt resembling a Masura pulse lentil in size, indicate the use of a rivet. Further, the text describing the material to be used to fabricate instruments mentions iron.[13] This mention of iron exclusively for instruments, vessels and scopes goes with the archeological evidence that points to India having moved directly to Iron Age without the intervening long stages of copper and bronze ages. The suggestion of non metallic materials like ivory indicates that iron technology was not widespread in remote areas and that the teacher was conscious of these facts. Moreover, this lends a clue to our surmise that the times of Sushrutha probably coincided with the early part of Iron Age in India. Various authorities fix these dates as being between 1000 and 500 BC.[14,15]

The reference to weather so graphically set out in the *Sushrutha Samhitha* mirrors the general pattern of the weather in north India.[16] Since the Indian ocean monsoon system has been thought to be in existence for more than 10,000 years, this is a strong indication that the origin of the text was from India, and with the supporting evidence based on the description of material we can safely assume that the text originated around 600BC, the earliest date mentioned by various authors.[17,18]

The absence of copper and bronze and exclusive use of iron in instruments and vessels lead us to conclude that the science of medicine as set out in the *Sushrutha Samhitha* evolved initially in post Harappan period; likely in an area of India where the first metal fabricated and used was iron. With the availability of only one text which has undergone many revisions, as the sole source, it is difficult to arrive at any firm conclusions in this regard.

The descriptions in *Sushrutha Samhitha* of the symptoms of the diseases and the prognosis are amazingly accurate. However, we know that surgical interventions had been attempted before by prehistoric men. The archeological evidence of trying to drain the large head in a hydrocephalus was reported from a late Paleolithic cave site at Rocheril in France, i.e. nearly 20,000 years ago.[18] All through human history man has striven to seek cures from the many diseases that afflicted him. In Chapter VIII, *Shastravacharaniyamadhyayam* (Instruments Used In Connection With a Surgical Operation); Sushrutha says *“A physician, skilled in the art of using surgical instruments, is always successful in his professional practice, and hence the practice of surgery should be commenced at the very outset of medical studies.”* This might reflect the fact that results of the surgeries performed were more predictable at that time than conditions where surgeries could not be performed.

That Sushrutha and other surgical teachers of the time spent considerable time and effort in evolving techniques for correction of loss of nose ear etc. with the use of local tissues as flaps, speaks volumes of their surgical genius and perspicacity. The exact level of knowledge of the detailed anatomy and physiology underlying these techniques cannot be deduced from the descriptions in the *Sushrutha Samhitha* alone, as this is primarily a “how to” text written as brief as possible. This is understandable as the labour required to produce such texts was immense and this did not significantly improve till the invention of the printing, another two thousand years later.

One problem in trying to decipher the text of *Sushrutha Samhitha* and other Ayurvedic texts was that having been written in abbreviated fashion as *slokas*, for ease of remembering, these texts needed the interpretation and explanation of an experienced teacher.[7] So while the core remains, subsequent generations may add information based on the evolving understanding of man and his diseases. This tendency is noticeable in the available English translations of the *Sushrutha Samhitha* also.[12]

While the achievements of *Sushrutha Samhitha* are commendable, the reasons for certain authors to fix Sushrutha to a specific caste need to be corrected to keep the historic narration form being influenced by unwanted prejudices.

As well described by various authors[1,7] the sequence of events leading to the discovery of Indian technique of nose reconstruction and its adoption in the west started a full one hundred years before the Bower manuscript came to light. These initial reporters were unaware of the history of, or existence of surgical traditions in India prior to this period. A full 100 years passed before the Bower manuscript, the only historic document on surgery in India came to light.[3]

Researchers[11] noted that the British were not aware of the Indian Rhinoplasty technique till as late as 1793. Mr. James Findlay and Mr. Thomas Crusoe, surgeons at the British Residency in Poona in 1793 witnessed the operation on “Cowasjee” and reported the details of the operation in the Madras Gazette and later in the Gentleman's magazine of London in October, 1794. The following line brings the potter into picture: “…….. when he had a new one put on by a man of the Brick maker (potter's) caste near Poona”.

Prof. P S Chari[7] states that the surgeon was mentioned, in one account, to be of the brick-maker's caste….” The remark “at the time of the operation, this artist - surgeon was the only one of his kind in India and that the art was hereditary in the family” might have lead to viewing Sushrutha and the “potter surgeon” to be from the same family.[1,7]

These descriptions amply demonstrate how the profile of Sushrutha had been made into that of a potter carrying on his ware in the dark bylanes of some obscure city. The only problem is that there was a time gap of 2500 years between the time of Sushrutha and the mysterious surgeon who operated on Cowasjee.

Moreover, the technique applied by the mysterious surgeon, who operated on Cowasjee, was different from that described in *Sushrutha Samhitha*.[7,11,19] Whereas Sushrutha used the superiorly based cheek flap the mysterious surgeon used the forehead flap. The confusion also stemmed from the two Sanskrit words *Kopal* which means cheek and *Kapal* which translates to forehead. Surely, in the intervening 2500 years, better flaps were developed! Thus while we can attribute confidently to Sushrutha the earliest use of Flap technique to reconstruct a tissue defect, the exact parentage of the forehead flap of Indian technique of nose reconstruction shall never be known with certainty.

*Sushrutha Samhitha* amply demonstrates that 2500 years ago there was a well structured surgical training programme, which is generally accepted. In selection of the student only the three castes of Brahmana, Kshatriya and Vysya are mentioned as admissible for training. Exceptionally, a Shudra could be admitted for training if he was intellectually promising.[20] Thus this was a period when no rigid caste restrictions were present. However this may throw light on reasons because of which the technique came to be followed more in familial traditions.

With this backdrop, we can well imagine how a western observer who had the occasion to experience the rigid caste barriers in vogue in Indian society then[21], on learning that the mysterious surgeon who operated on Cowasjee belonged to the caste of potters, might have erroneously inferred Sushrutha's caste therein; and further mistakenly asserted that the caste of potters were traditionally performing surgery in India (*Mathes, chapter 2).*[1] This view needs to be corrected. We should remember that absence of evidence is not evidence of absence.

## CONCLUSION

*Sushrutha Samhitha* as Bower manuscript is the earliest surviving script throwing light on the level of technology in surgery and medicine in the early history of India. This period can be reliably dated to be around 600 BC. By its very nature of transmission the root text was subject to various additions and alterations.

The text of the *Sushrutha Samhitha* itself indicates that this was a codification of then current practices, more in the nature of the lecture notes of today.

The techniques described in Sushrutha Samhitha are eminently in line with the technical abilities of the times. Technical refinement of surgical skills as in nose reconstruction were possible, practiced and thought of in those times.

The obvious description of the forehead flap, as performed by the mysterious surgeon and who operated on Cowasjee who belonged to the caste of potters, indicates that experimentation with and propagation of surgical skill present in the intervening 2500 years between Sushrutha and Cowasjee. This has to be presumed to have been transferred in the family.

Sushrutha's caste shall remain a mystery till documents of sufficient antiquity are revealed. Till such date, if indeed he has to have an epithet, it should rather be-‘Father of Surgery’.
